# Behavioural symptoms of attention deficit/hyperactivity disorder in preterm and term children born small and appropriate for gestational age: A longitudinal study

**DOI:** 10.1186/1471-2431-10-91

**Published:** 2010-12-15

**Authors:** Kati Heinonen, Katri Räikkönen, Anu-Katriina Pesonen, Sture Andersson, Eero Kajantie, Johan G Eriksson, Dieter Wolke, Aulikki Lano

**Affiliations:** 1 Institute of Behavioral Science, P.O. Box 9, FI-00014 University of Helsinki, Helsinki, Finland; 2 Institute of Clinical Medicine, University of Helsinki, Helsinki, Finland; 3 Hospital for Children and Adolescents, Helsinki University Central Hospital, Helsinki, Finland; 4 National Institute for Health and Welfare, Helsinki, Helsinki, Finland; 5 Department of General Practice and Primary Health Care, University of Helsinki, Helsinki, Finland; 6 Helsinki University Central Hospital, Unit of General Practice,Helsinki Finland; 7 Vasa Central Hospital, Vaasa, Finland; 8 Department of Psychology and HSRI, University of Warwick, Coventry UK

## Abstract

**Background:**

It remains unclear whether it is more detrimental to be born too early or too small in relation to symptoms of attention deficit/hyperactivity disorder (ADHD). Thus, we tested whether preterm birth and small body size at birth adjusted for gestational age are independently associated with symptoms of ADHD in children.

**Methods:**

A longitudinal regional birth cohort study comprising 1535 live-born infants between 03/15/1985 and 03/14/1986 admitted to the neonatal wards and 658 randomly recruited non-admitted infants, in Finland. The present study sample comprised 828 children followed up to 56 months. The association between birth status and parent-rated ADHD symptoms of the child was analysed with multiple linear and logistic regression analyses.

**Results:**

Neither prematurity (birth < 37 weeks of gestation) nor lower gestational age was associated with ADHD symptoms. However, small for gestational age (SGA < -2 standard deviations [SD] below the mean for weight at birth) status and lower birth weight SD score were significantly, and independently of gestational age, associated with higher ADHD symptoms. Those born SGA, relative to those born AGA, were also 3.60-times more likely to have ADHD symptoms scores above the clinical cut-off. The associations were not confounded by factors implicated as risks for pregnancy and/or ADHD.

**Conclusions:**

Intrauterine growth restriction, reflected in SGA status and lower birth weight, rather than prematurity or lower gestational age *per se*, may increase risk for symptoms of ADHD in young children.

## Background

Prematurity and small body size at birth are associated with increased risk for attention deficit/hyperactivity disorder (ADHD) or its symptoms [1-4]. This risk may be particularly characteristic to those born small for gestational age (SGA) [1,5-7]. Yet, there is evidence that these associations may rather be depicted by a dose-response relationship across the whole range of gestational age [8] and birth weight adjusted for gestational age [9]. Consequently, it remains unclear whether it is more detrimental to be born too early or too small. As the biological basis of lower gestational age and retarded fetal growth differ [10,11] disentangling effects of these two might offer insight into the prenatal origins of ADHD. Accordingly, the major aim of this study was to explore the long-term independent and interactional effects of premature *versus *term birth and of SGA *versus *appropriate for gestational age (AGA) status on behavioural symptoms of ADHD in a well-characterized sample of 828 Finnish children followed up to 56 months of age.

## Methods

### Participants

**Figure 1:**
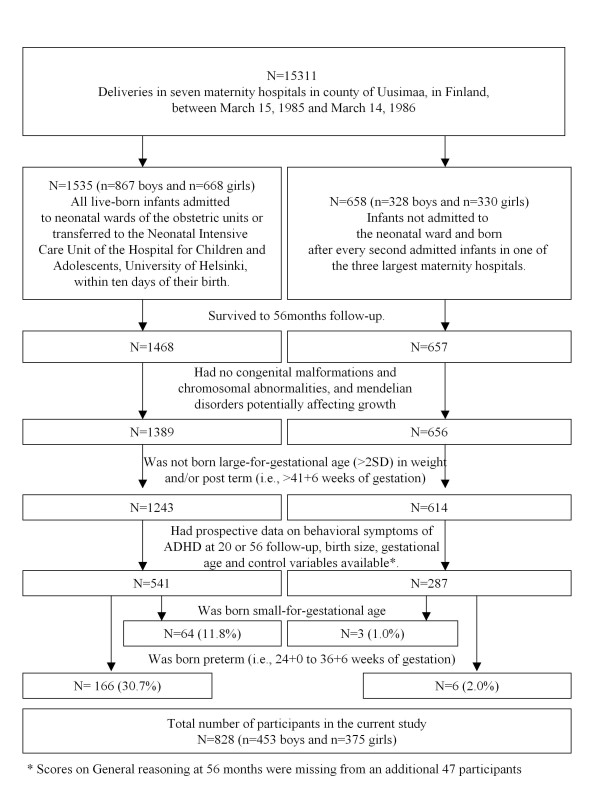
**Participants of the study**.

The study cohort comprised a subsample of 2193 infants participating in the Arvo Ylppö Longitudinal Study (AYLS) [12,13]. Figure 1 presents the selection of the participants. The present study included 828 boys (n = 453) and girls (n = 375) who did not had congenital malformations, chromosomal abnormalities or mendelian disorders potentially affecting growth, and who were not born large-for-gestational age or post-term and had prospective data available up to 56 months. The study protocol was approved by the ethics committees of the participating hospitals, and the parent(s) gave their informed consent.

The study participants (n = 828) did not differ from those who were lost to follow-up due to lack of information on birth weight, gestational age or pre- and neonatal control variables (N = 88), and/or additionally due to lack of parent rated symptoms of ADHD (n = 941) in sex, multiple pregnancy, mother's age, height, BMI at the end of pregnancy or change in weight during pregnancy, in parental education or in admission to neonatal ward (P-values > 0.05). Further, those lost to follow-up due to lack of parent rated ADHD symptoms did not differ from the participants in the current study in gestational age/preterm status or in birth weight standard deviations [SD]/SGA status (P-values > 0.13). However, those lost to follow-up due to lack of information on pre- and neonatal variables were more likely to have been admitted to a neonatal ward, to come from less educated families, and their mothers smoked more during pregnancy (*P'*s < 0.02). Whereas, those lost to follow-up due to lack of parent rated ADHD symptoms had mothers who smoked more during pregnancy (P = 0.003).

### Measures

#### Gestational age

Gestational age based on ultrasound examination was available for 671 participants (81.0%); for the remaining 157 (19.0%), gestational age was calculated from the date of the mother's last menstrual period. In addition, all infants were given a Dubowitz assessment of gestational age [14]. When there was a difference in the estimates of >2 weeks, the Dubowitz assessment results were used, except for preterms under 32 weeks of gestational age [13]. Of the 828 children, 172 (20.8%) were born preterm (24+0 to 36+6 weeks of gestation), and the remaining children at term (37+0 to 41+6 weeks of gestation).

#### Birth weight

Data on the newborns' weight (g) were extracted from birth records. Birth weight was transformed into gestational age -adjusted standard deviation (SD) scores according to Finnish growth charts. Children born < -2 SDs of the mean for weight at birth (n = 67, 8.1%) were classified as SGA [15]. The remaining children, ≥-2 SDs of the mean but <2 SDs of the mean, were classified as AGA. SGA group included both preterm (n = 28) and term (n = 39) born children.

#### Parental Ratings of ADHD symptoms

At the 56-month follow-up the parents evaluated the child's behavioural symptoms of ADHD with the Conners' Hyperactivity Index-parent version [16]. This index is composed of ten items rated on a four-point scale (0 = not at all to 3 = very much). Sample items are:"Inattentive, easily distracted," "Restless or overactive," "Fails to finish things he/she starts," "Fidgeting" and "Demands must be met immediately - easily frustrated." The items were summed with higher scores reflecting higher levels of behavioural symptoms of ADHD (Mean = 8.97, SD = 4.89, range: 0 to 30). Cronbach's alpha was 0.85. In addition, to treating the Conners' Hyperactivity Index as a continuous measure, a score of > 15 was used to identify those above the clinically significant cut-off of symptoms [16] (n = 69, 8,3% had scores above the clinical cut-off).

#### Potential confounders

We tested if the effects of preterm *versus *term birth, and of SGA *versus *AGA status on ADHD symptoms were confounded by the child's sex, multiple pregnancy (singleton vs. multiple), mother's smoking during pregnancy (none, 1-10, >10 cigarettes/day), parental education (four point scale of highest self-reported level of education of either parent: from high = university education to low = elementary school education or less), maternal age (yrs), maternal height (cm), maternal body mass index (kg/m2) at the end of pregnancy and change in weight (kg) during the pregnancy. In addition, as symptoms of ADHD and cognitive performance are associated [17] we analyzed also whether the associations were confounded by the child's general reasoning measured at the age of 56 months with Columbia Mental Maturity-scale (CMMS) [18,19]. The CMMS is a non-verbal cognitive ability test consisting of pictorial and figural classification items. The child has to select from sets of 3-5 drawings one drawing that is different or unrelated to the others. The reliability of CMMS is high [12] and it has been shown to be a valid assessment of nonverbal intelligence quotient [18,20].

### Statistical analysis

As the primary data analytical tools, we used multiple linear regression analysis (symptoms of ADHD as continuous measure) and logistic regression analysis (symptoms of ADHD as dichotomous: > 15 points as clinical cut-off) [16]. We examined differences in ADHD symptoms at 56 months of age between children born (1) preterm and term, and (2) SGA and AGA. We tested these associations, first, after controlling the sex of the child; second, after controlling for pre- and neonatal confounders; and third after controlling child's general reasoning at 56 months of age.

To test whether any potential differences in ADHD symptoms of children born preterm and term, and SGA and AGA were independent of each other, both these dichotomous variables were entered simultaneously into the regression equation. To test for interactional effects, an interaction term 'preterm/term status × SGA/AGA status' was entered into regression equation in addition to the main effects of these two dichotomous variables.

Further, to test whether associations differed between boys and girls an interaction term 'sex × preterm/term', and 'sex × SGA/AGA-status' was entered into regression equation in addition to the main effects of these variables.

All analyses were also run using gestational age and birth weight SD scores as continuous variables.

## Results

Characteristics of the sample according to preterm *versus *term birth and SGA *versus *AGA status are presented in Tables 1 and 2. Before proceeding to analyses targeting the major study questions, we tested associations between ADHD symptoms of the child and the potential confounding variables. Compared to girls, boys had higher parent-rated ADHD symptoms scores (Mean difference [MD] = 1.18, P < 0.001). After controlling for sex, the ADHD symptoms scores were also higher for children with a lower parental level of education (P = 0.001), whose mothers' were younger at delivery (P = 0.01), and who scored lower in the general reasoning test at 56 months of age (P < 0.001).

**Table 1:** Characteristics of the sample by term and preterm birth.

	Term (n = 656)	Preterm (n = 172)		
	Mean (SD)/	Mean (SD)/		
	n (%)	n (%)	p1	p2
Prenatal and maternal characteristics				
Girl (n/%)	302 (46.0)	73 (42.4)	0.40	0.38
Multiple birth (n/%)	15 (2.3)	29 (16.9)	< 0.001	< 0.001
Mother's smoking during pregnancy (n/%)				
> 10	39 (5.9)	9 (5.2)	0.90	0.52
1-10	104 (15.9)	39 (22.7)	0.04	0.07
none	513 (78.2)	124 (72.1)	-	-
Mother's age (years)	29.32 (5.1)	29.65 (5.2)	0.45	0.53
Mother's height (cm)	164.18 (5.3)	164.29 (5.6)	0.80	0.50
Mother's BMI at the end of pregnancy (m2/kg)	27.55 (4.1)	26.26 (3.5)	< 0.001	< 0.001
Mother's change in weight during pregnancy (kg)	11.83 (4.5)	9.98 (4.9)	< 0.001	< 0.001
Birth characteristics				
Birth weight (grams)	3513 (533)	2282 (604)	-	< 0.001
Gestational age (weeks)	39.4 (1.3)	34.0 (2.4)	-	< 0.001
Birth weight standard deviation score	0.0 (1.1)	-0.66 (1.3)	-	< 0.001
Parental educational attainment (n/%)				
High	153 (23.3)	38 (22.1)	-	-
Upper middle	187 (28.5)	49 (28.5)	0.83	0.76
Lower middle	240 (36.6)	61 (35.5)	0.92	0.92
Low	76 (11.6)	24 (14.0)	0.42	0.53
Child's General Reasoning at 56 months*	100.0 (17.2)	97.7 (16.6)	0.14	0.14

Note. p1 = Unadjusted associations, p2 = Adjusted for SGA vs. AGA status. * Missing values, N = 47

**Table 2:** Characteristics of the sample by AGA and SGA status.

	AGA (n = 761)	SGA (n = 67)		
	Mean (SD)/	Mean (SD)/		
	n (%)	n (%)	p1	p2
Prenatal and maternal characteristics				
Girl (n/%)	344 (45.2)	31 (46.3)	0.87	0.76
Multiple birth (n/%)	27 (3.5)	17 (25.4)	< 0.001	< 0.001
Mother's smoking during pregnancy (n/%)				
> 10	38 (5.0)	10 (14.9)	< 0.001	0.001
1-10	128 (16.8)	15 (22.4)	0.15	0.20
none	595 (78.2)	42 (62.7)	-	
Mother's age (years)	29.88 (5.2)	29.34 (5.1)	0.41	0.47
Mother's height (cm)	164.34 (5.4)	162.51 (4.7)	0.01	0.01
Mother's BMI at the end of pregnancy (m2/kg)	27.32 (4.0)	26.83 (3.7)	0.33	0.68
Mother's change in weight during pregnancy (kg)	11.51 (4.64)	10.73 (4.4)	0.19	0.53
Birth characteristics				
Birth weight (grams)	3363 (670)	2054 (366)	< 0.001	-
Gestational age (weeks)	38.4 (2.7)	37.0 (2.5)	< 0.001	-
Birth weight standard deviation score	0.1 (0.9)	-2.7 (0.5)	< 0.001	-
Parental educational attainment (n/%)				
High	178 (23.4)	13 (19.4)	-	-
Upper middle	223 (29.3)	13 (19.4)	0.58	0.55
Lower middle	271 (35.6)	30 (44.8)	0.23	0.23
Low	89 (11.7)	11 (16.4)	0.22	0.27
Child's General Reasoning at 56 months*	99.6 (17.1)	99.1 (17.1)	0.82	0.99

Note. p1 = Unadjusted associations, p2 = Adjusted for term vs. preterm birth. * Missing values, N = 47

### Birth status and parent-rated behavioural symptoms of ADHD

Table 3 shows that preterm and term children did not differ significantly from each other in parent-rated behavioural symptoms of ADHD (tested either as continuous or dichotomous). Gestational age as a continuous variable was also not significantly associated with ADHD symptoms of the child (all P-values >.36 for ADHD symptoms as continuous or dichotomous; data not shown).

**Table 3:** ADHD symptoms among 56 months old children born term and preterm.

	Term vs. Preterm			
	ADHD symptoms as continuous		ADHD symptoms as dichotomous	
	B (95% CI)	P-value	Odds ratio (95% CI)	P-value
Model 1	0.27 (-0.55 to 1.09)	0.52	1.15 (0.64 to 2.08)	0.63
Model 2	0.21 (-0.66 to 1.07)	0.64	1.13 (0.60 to 2.13)	0.71
Model 3	-0.06 (-0.94 to 0.82)	0.90	1.06 (0.53 to 2.10)	0.87
Model 4	-0.15 (-1.03 to 0.73)	0.74	0.91 (0.45 to 1.84)	0.80

Note. Model 1: Adjusted for child's sex; Model 2: Adjusted for Model 1 + multiple pregnancy, mother's smoking during pregnancy, parental education, maternal age, maternal height, maternal body mass index at the end of pregnancy and change in weight during the pregnancy; Model 3: Adjusted for Model 2+child's general reasoning at 56 months old; Model 4: Adjusted for Model 3 + SGA vs. AGA status.

Table 4 shows that in comparison to children born AGA, those born SGA earned higher ADHD symptoms scores. Further, those born SGA (n = 13 out of 67, 19.4%), relative to those born AGA (n = 56 out of 761, 7.4%), were 3.60 times more likely to have ADHD symptoms scores above the clinical cut-off (Table 4). Birth weight as a continuous SD score showed similar findings: for every standard deviation decrease in birth weight, ADHD symptoms scores increased by 0.38 points (95% CI: -0.71 to -0.06, P < 0.02 for a fully adjusted model), and the risk for the children to have ADHD symptoms scores above the clinical cut-off increased by 1.51 -times (95% CI: 1.18 to 1.93, P = 0.001 for a fully adjusted model).

**Table 4:** ADHD symptoms among 56 months old children born AGA and SGA.

	AGA vs. SGA			
	ADHD symptoms as continuous		ADHD symptoms as dichotomous	
	B (95% CI)	P-value	Odds ratio (95% CI)	P-value
Model 1	1.68 (0.47 to 2.90)	0.006	3.05 (1.57 to 5.94)	0.001
Model 2	1.77 (0.51 to 3.04)	0.006	3.18 (1.54 to 6.55)	0.002
Model 3	1.52 (0.22 to 2.82)	0.02	3.53 (1.62 to 7.68)	0.001
Model 4	1.54 (0.24 to 2.84)	0.02	3.60 (1.63 to 7.95)	0.002

Note. Model 1: Adjusted for child's sex; Model 2: Adjusted for Model 1 + multiple pregnancy, mother's smoking during pregnancy, parental education, maternal age, maternal height, maternal body mass index at the end of pregnancy and change in weight during the pregnancy; Model 3: Adjusted for Model 2+child's general reasoning at 56 months old; Model 4: Adjusted for Model 3 + preterm vs. term status

There were no significant 'preterm/term- × SGA/AGA-status' or 'gestational age × birth weight SD score' - interactions (P-values > 0.07). Neither were there any significant sex-specific associations (all P-values > 0.17 for 'sex × preterm/term', sex × gestational age', 'sex × SGA/AGA' and 'sex × birth weight SD score'-interactions).

Finally, the analyses were re-run excluding children from multiple pregnancies (n = 44). The results remained virtually identical (all P-values > 0.20 for association between ADHD symptoms and SGA/AGA-status/gestational age, and all P-values < 0.01 for associations between ADHD symptoms and term/preterm/birth weight SD score).

## Discussion

The present study showed that preterm birth (before 37+0 weeks of gestation) was not associated with higher parent-rated behavioural symptoms scores of ADHD at 56 months of age. However, being born SGA (< -2 SD) was associated with higher ADHD symptoms scores. Those born SGA, relative to those born AGA, were also over three times more likely to have ADHD symptoms scores above a cut-off indicative of a clinically significant level of symptoms. The findings were similar when gestational age and birth weight SD score were used as continuous variables: gestational age was not, while lower birth weight SD was associated with higher ADHD symptoms scores and with scores that were above the clinical cut-off. The effects of SGA status and birth weight SD score were independent of prematurity and gestational age. The effects were also not confounded by parent- or child-related characteristics that may pose a risk for pregnancy and/or ADHD or its symptoms. Our findings, thus, suggest that intrauterine growth restriction, as reflected in the SGA status and lower birth weight SD score, rather than prematurity or lower gestational age *per se*, may pose a risk for symptoms of ADHD in young children.

The current results are in line with previous findings suggesting that the children born SGA rather than those born AGA, may be the most vulnerable for displaying symptoms of ADHD later in life [1,5-7]. However, our findings strengthen and extend the previous ones in significant ways by suggesting that the risk for ADHD symptoms is not merely confined to the SGA children born < 1500 g [1] or term [5,7], but that the effects of SGA status and lower birth weight appear similar across the whole range of gestational age [6]. To our knowledge, earlier studies have also not addressed the independent effects of prematurity/gestational age and SGA status/birth weight SD, and/or tested the interaction between them in predicting symptoms of ADHD. In contrast to previous findings [1-4,8], we did not find associations from prematurity to symptoms of ADHD. The current study differs from previous studies which have analyzed either extremely premature/low birth weight children [1-4] or used clinically verified disorder as the outcome measure [8], and thus is not directly comparable to the previous studies. Previous studies identified Attention Deficit rather than ADHD symptoms to be associated to severe prematurity [21,22]. Further, we had relative small number of the most extremely premature born children and the current study may thus lack the statistical power to detect the previously shown association.

The major strength of this study was the inclusion of well-defined gestational age and birth weight SD scores as dichotomous and continuous variables. Further, a number of parental- and child- related variables have been implicated as risks for pregnancy and/or symptoms of ADHD of the child. In the current study we could control for several of those factors. We also had extensive clinical data and thus could exclude children with severe neonatal or childhood conditions that could have potentially confounded the findings.

There are some limitations to the study. For practical constraints several parents did not receive questionnaire of the child's ADHD symptoms at the 56 months follow-up or failed to fill it in (N = 941 of those who did have all pre- and neonatal variables used in this study available). However, the participants in the present study did not differ from those lost to follow-up due to lack of information on ADHD symptoms, except that their mothers smoked less during pregnancy. Yet, there might be other unmeasured or unknown social factors that might have affected the five year outcome. In the light of the current confounders the found results may be more characteristic of children developing in more affluent prenatal environments.

Further, behavioural symptoms of ADHD were evaluated only by the parent and we did not have any information of the child's behaviour in different settings e.g., in day care. However, in Finland only approximately half of the children aged 3-5 were in a full-time day care in the nineties http://www.sotkanet.fi and school does not start before age of seven. Finally, we did not have any information on the parental behavioural symptoms of ADHD and were thus unable to adjust for the possible genetic susceptibility.

The underlying mechanisms explaining the association might be either biological or psychosocial, or both. Causes of both preterm birth and intrauterine growth restriction (IUGR) are multiple. Preterm labour/birth may be initiated by infection or inflammation, uteroplacental ischaemia or haemorrhage, uterine overdistension, stress, and other immunologically mediated processes [10]. Even though born immature, (e.g. at 35 weeks of gestation, the weight of the brain is only 60% of that at term), preterm infants might have grown optimally during the fetal period. SGA born infants' foetal environment and growth, however, have been less optimal. Fetal growth restriction may result from e.g. foetal and maternal genetic variations, fetal chromosomal anomalies, placental anomalies, maternal environment (e.g., low pre-pregnancy weight), mother's inadequate energy and protein intake during pregnancy, from maternal health behaviours (e.g., smoking), disorders (e.g., hypertension), and plasma volume expansion [11]. However, in the current study the children with fetal chromosomal anomalies were excluded, and thus it is not a potential explanation of the findings in this particular sample.

Evidence exists also that brain structure differs in growth restricted infants. It has been shown for example that term-SGA children have a reduced total brain volume [23], and preterm-SGA infants have a significant reduction in absolute cortical gray matter volume, in overall brain tissue volumes [24] and in hippocampal volume [25]. Further, a smaller total brain volume has been shown to be associated with diagnosed ADHD [26] and the reduced gray matter volumes with immature attention-interactional scores [24]. Furthermore, in addition to potentially influencing the structural development of the brain, early life adversities may also be reflected in an imbalance in the function of noradrenergic and dopaminergic systems that have shown to play a role in ADHD [27]. Finally, we cannot rule out the possibility that ADHD, which is a highly heritable disorder [28], shares a common genetic origin with SGA status.

Mothers with low birth weight children are faced with challenges that impact their parenting and perceptions of their child [29]. Mothers of children in special care have negative recollections of birth affecting their behaviour with their child [30]. Early experiences and perceptions in turn can predict compromised parenting and impact child's development [30]. Mothers who see their children as vulnerable feel less in control of their children's behaviour and provide less stimulating and less positive experiences to their child [31]. In the current study behavioural symptoms of ADHD were evaluated only by the parent. Parent's evaluation may thus been biased due to challenges that small and preterm children are faced, and these biases may have, in turn, affected the child's development.

## Conclusions

Our study extends the existing literature by showing that intrauterine growth restriction, reflected in SGA status and lower birth weight SD score, rather than prematurity or lower gestational age *per se*, may increase risk for symptoms of ADHD in young children. Future studies are needed to replicate these findings and clarify mechanisms underlying the found associations.

## Competing interests

The authors declare that they have no competing interests.

## Authors' contributions

KH and KR made substantial contributions to analysis and interpretation of data and have been involved in drafting the manuscript and revising it critically for important intellectual content. A-KP, SA, EK, EGJ have contributed to analysis and interpretation of data, and have been involved in revising manuscript critically for important intellectual content; DW has made substantial contributions to conception and design, and acquisition of data as well as to interpretation of data, and has revised manuscript critically for important intellectual content. AL has made substantial contributions acquisition of data and has been involved in revising manuscript critically for important intellectual content. All authors read and approved the final manuscript.

## Pre-publication history

The pre-publication history for this paper can be accessed here:

http://www.biomedcentral.com/1471-2431/10/91/prepub
